# MicroRNA-146a Regulates Perfusion Recovery in Response to Arterial Occlusion *via* Arteriogenesis

**DOI:** 10.3389/fbioe.2018.00001

**Published:** 2018-01-22

**Authors:** Joshua L. Heuslein, Stephanie P. McDonnell, Ji Song, Brian H. Annex, Richard J. Price

**Affiliations:** ^1^Department of Biomedical Engineering, University of Virginia, Charlottesville, VA, United States; ^2^Robert M. Berne Cardiovascular Research Center, University of Virginia, Charlottesville, VA, United States

**Keywords:** microRNA, peripheral arterial disease, angiogenesis, arteriogenesis, endothelial cell, shear stress, epigenetics, hindlimb ischemia

## Abstract

The growth of endogenous collateral arteries that bypass arterial occlusion(s), or arteriogenesis, is a fundamental shear stress-induced adaptation with implications for treating peripheral arterial disease. MicroRNAs (miRs) are key regulators of gene expression in response to injury and have strong therapeutic potential. In a previous study, we identified miR-146a as a candidate regulator of vascular remodeling. Here, we tested whether miR-146a regulates *in vitro* angiogenic endothelial cell (EC) behaviors, as well as perfusion recovery, arteriogenesis, and angiogenesis in response to femoral arterial ligation (FAL) *in vivo*. We found miR-146a inhibition impaired EC tube formation and migration *in vitro*. Following FAL, Balb/c mice were treated with a single, intramuscular injection of anti-miR-146a or scramble locked nucleic acid (LNA) oligonucleotides directly into the non-ischemic gracilis muscles. Serial laser Doppler imaging demonstrated that anti-miR-146a treated mice exhibited significantly greater perfusion recovery (a 16% increase) compared mice treated with scramble LNA. Moreover, anti-miR-146a treated mice exhibited a 22% increase in collateral artery diameter compared to controls, while there was no significant effect on *in vivo* angiogenesis or muscle regeneration. Despite exerting no beneficial effects on angiogenesis, the inhibition of mechanosensitive miR-146a enhances perfusion recovery after FAL *via* enhanced arteriogenesis.

## Introduction

Peripheral arterial disease (PAD) is caused by blockage(s) of the arteries, in the lower limbs, due to occlusive atherosclerosis (Norgren et al., 2007). There are no medical therapies available to treat PAD and many PAD patients are not amenable to surgical revascularization options or receive little long-term benefit from such surgeries (Annex, 2013). This has led to the rise of new therapeutic strategies that have sought to induce endogenous revascularization *via* stimulation of new capillary growth from preexisting vessels (i.e., angiogenesis) and/or lumen expansion of preexisting arteries (i.e., arteriogenesis) to restore lower limb perfusion. Yet, large-scale therapeutic clinical trials to this end have been largely unsuccessful to date (Seiler et al., 2001; van Royen et al., 2005; Zbinden et al., 2005; Ripa et al., 2006; Subramaniyam et al., 2009). These failures highlight our incomplete understanding of the basic mechanisms of revascularization and underscore the critical need for the continued study of the endogenous regulation of this complex, highly orchestrated process.

MicroRNAs (miRs) have emerged as key regulators of the response to injury including vascular remodeling, comprising of both angiogenesis (Urbich et al., 2008; Neth et al., 2013; Santulli, 2016) and arteriogenesis (Welten et al., 2014; Ganta et al., 2017; Guan et al., 2017). In addition, we reported recently that collateral artery segments exhibit either “moderate” or “amplified” arteriogenesis depending on the hemodynamics to which they are exposed (i.e., non-reversed or reversed flow waveforms, respectively) following femoral arterial ligation (FAL) (Heuslein et al., 2015b). By applying shear stress waveforms biomimetic of these *in vivo* hemodynamics to endothelial cells (ECs) *in vitro*, we identified several mechanosensitive miRs (-100, -199a, and -146a,) as potential negative regulators of arteriogenesis. To this end, inhibition of miR-100 has been shown previously to enhance perfusion recovery following FAL (Grundmann et al., 2011), and we have recently determined that miR-199a inhibition is a potent enhancer of perfusion recovery and arteriogenesis following FAL in Balb/c mice (Heuslein and Price, 2017). Though miR-146a has been shown to be enriched in ECs *in vivo* (Cheng et al., 2013) and to regulate both endothelial activation (Cheng et al., 2013) and *in vitro* angiogenesis (Zhu et al., 2013, 2016; Rau et al., 2014), the role of miR-146a following FAL is unknown. Here, we tested the hypothesis that miR-146a regulates angiogenesis, arteriogenesis, and perfusion recovery after FAL.

## Materials and Methods

### Human Umbilical Vein Endothelial Cell (HUVEC) Culture

Human umbilical vein endothelial cells, purchased from Cell Applications Inc. (San Diego, CA, USA), were thawed and maintained on tissue culture treated flasks in complete endothelial growth medium (Cell Applications). For *in vitro* transfection experiments, HUVECs were transfected at 30 nM for 24 h with miRVana anti-miR-146a (#446084, ThermoFisher Scientific, Waltham, MA, USA) or miR inhibitor negative control (#4464076, ThermoFisher Scientific) using siPORT NeoFX transfection agent (#AM4510, ThermoFisher Scientific) according to the manufacturer’s instructions. Twenty-four hours after transfection, cell culture medium was changed. For HUVECs cultured under normal growth condition, culture medium was changed to normal growth medium containing 10% fetal bovine serum (FBS) and cells were incubated for the given experimental time at normoxia conditions (20% O_2_). For HUVECs cultured under hypoxia serum starvation (HSS) to simulate ischemia *in vitro*, culture medium was changed to endothelial serum starvation medium (#209-250, Cell Applications) and cells were incubated in a 2% oxygen chamber (BioSpherix, Lacona, NY, USA) for the given experimental duration. For each set of experimental comparisons, cells were used between subculture passages 4 and 7.

### Endothelial Matrigel Assay

Following transfection, HUVECs were plated on growth factor reduced Matrigel^®^ (#356231, BD BioSciences, Bedford, MA, USA). A chilled 48-well plate was coated with thawed Matrigel^®^ (200 μL/well) then incubated at 37°C for 30 min to allow gel to form. HUVECs were then plated at 30,000 cells/well and exposed to either normal growth or HSS conditions for 6 h. The 6-h duration was chosen based on similar studies examining formation of capillary-like structures (Hazarika et al., 2013; Rau et al., 2014; Wang et al., 2015). Three fields of view were captured per image using a 4× objective on an Olympus IX50 microscope. Number of loops (i.e., number of meshes or enclosed network of tubes) and number of tubes were quantified using the ImageJ plugin “Angiogenesis Analyzer”.

### EC Survival

Human umbilical vein endothelial cells were plated at 1 × 10^4^ cells/well in a 96-well plate. Similar to previous studies in our lab (Hazarika et al., 2013; Wang et al., 2015), following transfection, cells were exposed to normal growth conditions (10% FBS, 20% O_2_) or HSS conditions for an additional 24 h. During the final 4 h of incubation, tetrazolium salt WST-1/ECS solution (#K301, BioVision, Milpitas, CA, USA) was added to culture medium. At 24 h, cell viability was assessed as the resulting tetrazolium salt cleavage to formazan by mitochondrial dehydrogenases.

### EC Permeability

Human umbilical vein endothelial cells were plated and grown until confluent in a 6-well Transwell plate with 0.4-µM pores (#3450, Corning, Tewksbury, MA, USA). Following transfection, HUVECs were cultured in serum-free medium without phenol red and exposed to HSS conditions for an additional 24 h. Rhodamine dextran (1 mg/mL) was added to the HUVEC medium (top Transwell chamber) and HUVECs incubated for an additional 30 min. Following incubation 100 μL samples were drawn from the bottom chamber in triplicate and fluorescence was read at 540ex/615em.

### Endothelial Scratch Assay

Human umbilical vein endothelial cells were plated at ~4 × 10^4^ cells/well and grown until confluent. Following transfection, cells were scratched with a p200 pipette tip then washed twice. HUVECs were then cultured in normal growth or HSS conditions for 12 h. Images were captured using a 4× objective on an Olympus IX50 microscope.

### Mice

This study was carried out in accordance with the recommendations of the American Heart Association Guidelines for the Use of Animals in Research. The protocol was approved by the Institutional Animal Care and Use Committee at the University of Virginia (Protocol 3814).

Balb/c mice were purchased from Charles River Laboratory (Wilmington, MA, USA). All animals were housed in the animal facilities at the University of Virginia.

### FAL Model

We used a previously detailed FAL scheme (Meisner et al., 2012; Heuslein et al., 2015b, 2016, 2017) that produces consistent arteriogenesis in the collateral arteries of the gracilis adductor muscles (Chappell et al., 2008; Distasi et al., 2009; Nickerson et al., 2009a; Dai and Faber, 2010; Meisner et al., 2013, 2014; Heuslein et al., 2015b, 2016, 2017), along with minimal heterogeneity in the baseline collateral structure with known changes in flow direction from baseline. Male mice, 10–12 weeks of age, were anesthetized (i.p. 120 mg/kg ketamine, 12 mg/kg xylazine, and 0.08 mg/kg atropine), depilated, and prepped for aseptic surgery. On the left leg, an incision was made directly above and along the femoral artery, which was gently dissected from the femoral vein and nerve between the bifurcation of the superior epigastric artery and popliteal artery. Two 6.0 silk sutures were placed immediately distal to the epigastric artery, which served as the origin of the muscular branch artery in all mice, and the artery segment between the two ligatures was then severed with micro dissecting scissors. The surgical site was then closed with 5.0 prolene sutures. A sham surgery, wherein the femoral artery was exposed but not ligated, was performed on the right hindlimb (i.e., on the other leg). Animals received one injection of buprenorphine for analgesia at the time of surgery and a second dose 8–12 h later.

### *In Vivo* miR-146a Antagomir Treatment

Anti-miR-146a-5p (5′-TGGAATTCAGTTCTC-3′) and non-targeting control (5′-ACGTCTATACGCCCA-3′) locked nucleic acid (LNA) oligonucleotides were purchased from Exiqon. Oligonucleotides were reconstituted in sterile TE buffer and stored at 1.2 nmol/µL at −20°C. Prior to use, aliquots of oligonucleotides were thawed and diluted in sterile saline to a final concentration of 0.25 nmol/µL. Immediately following FAL, 7.5 nmol of oligonucleotide was injected into both (ligated and sham-operated) gracilis (thigh) muscles.

### Laser Doppler Perfusion Imaging

For monitoring blood flow recovery and post-surgical ischemia, mice were anesthetized *via* 1.5% isoflurane under constant oxygen. Mice were placed in a prone position and the soles of the feet were scanned (PeriCam PSI, PeriMed, Stockholm, Sweden). Mean foot perfusion was used to calculate relative perfusion ratio (ligated/unligated).

### Tissue Harvesting for miR and Target Expression

Seven days after FAL, mice were anesthetized (i.p. 120 mg/kg ketamine, 12 mg/kg xylazine, and 0.08 mg/kg atropine) and euthanized *via* an overdose of pentobarbital sodium and phenytoin sodium (Euthasol^®^, Virbac, Fort Worth, TX, USA). The left ventricle was cannulated with a 23-G catheter (right ventricle was carefully opened to act as a sink for perfusate), and the entire body was perfused with 7 mL of Tris–CaCl_2_ (0.1 g/L CaCl_2_) containing 2% heparin, 2 mmol/L adenosine (16404, Fisher Scientific, Pittsburg, PA, USA), and 0.1 mmol/L papaverine (P3510, Sigma-Aldrich, St. Louis, MO, USA) to clear and vasodilate the downstream vasculature at a constant rate of 1.5 mL/min (PHD2000, Harvard Apparatus). Once perfused, we waited 5 min to enable vasodilation. The entire body was then perfused with 14 mL Tris–CaCl_2_. Both gracilis and gastrocnemius muscles, as well as the liver, spleen, and kidneys were dissected free, placed in RNAlater (Ambion), and stored at −20°C.

### RNA Isolation from Tissues and qRT-PCR

Excess RNAlater was removed from tissues. Tissues were then incubated in 450 µL TRIzol reagent for 5 min at room temperature. Tissues were then placed on ice and homogenized using a power homogenizer (Omni International, Kennesaw, GA, USA) in short bursts to avoid overheating. Following homogenization, an additional 550 µL TRIzol reagent was added. Samples were incubated for another 5 min at room temperature to ensure complete lysis. 200 µL of chloroform was added to each sample. Samples were then shaken vigorously for 15 s and incubated for 3 min at room temperature. Following this incubation, samples were centrifuged for 10 min at 12,000 *g* at 4°C. The resulting aqueous layer was carefully removed, place in a new RNA/DNAse-free tube, and an equal volume of 70% ethanol was added to the save aqueous layer. RNA isolation then proceeded using the PureLink total RNA purification system (Life Technologies Inc.) with the on-column DNase protocol (Life Technologies Inc.) according to the manufacturer’s instructions. RNA concentration and purity was determined with a NanoDrop spectrophotometer in duplicate.

For miR reverse transcription (Applied Biosystems, #4366596), miR-146a-5p (#000468), miR-539-5p (#001286), and miR-219c-3p (#466357_mat) detection, TaqMan MicroRNA assays (Applied Biosystems) were used. For mRNA reverse transcription, the SuperScript III First Strand Synthesis Super mix (#11752-250), ThermoFisher Scientific was used. TaqMan qPCR primers were used for *Card10* (Mm00459941), *Irak1* (Mm01193538), *Irak2* (Mm01184677), *Smad4* (Mm03023996), and *Nos3* (Mm00435217) qPCR. All quantitative real-time PCRs were done on a BioRad CFX96 detection system using the Bioline SensiMix II Probe Kit (#BIO-83005, Bioline, London, UK). miR expression was normalized to sno-202 (Applied Biosystems, #001232), whereas mRNA expression was normalized to *Hprt* expression (Mm03024075) and the relative expression was determined with the comparative 2^ΔΔCt^ method.

### Tissue Harvesting for Whole Mount Vascular Casting and Cross-sectional Analysis

For analysis of lumen diameters in the gracilis collateral arteries and to enable sectioning at specific regions, vascular casting was performed using an opaque polymer that allows for accurate lumen diameter measurements (Distasi et al., 2009). Twenty-one days after FAL, mice were anesthetized (i.p. 120 mg/kg ketamine, 12 mg/kg xylazine, and 0.08 mg/kg atropine), euthanized *via* an overdose of pentobarbital sodium and phenytoin sodium (Euthasol^®^), and then the abdominal aorta was cannulated. The lower body was then perfused with 7 mL of 2% heparinized saline with 2 mmol/L adenosine (16404, Fisher Scientific) and 0.1 mmol/L papaverine (P3510, Sigma-Aldrich) to clear and vasodilate the downstream vasculature at a constant rate of 1 mL/min (PHD2000, Harvard Apparatus). Once perfused, we waited 5 min to enable vasodilation. Tissues were then perfused with 3 mL of 4% paraformaldehyde solution (19943, Affymetrix, Cleveland, OH, USA) at 1 mL/min and allowed to fix for 10 min. The lower body was then perfused with 0.8 mL Microfil^®^ casting agent (FlowTech, Inc., Carver, MA, USA) at a constant speed of 0.15 mL/min. The viscosity of Microfil^®^ was adjusted to minimize transport across capillaries. After curing for 1.5 h at room temperature, gracilis muscles were dissected free and then cleared in 50% glycerol in phosphate-buffered saline overnight. Cleared tissues were mounted between two coverslips using 500-µm thick spacers (645501, Grace Bio-Labs Inc.) to keep constant thickness between muscles. Muscles were imaged using transmitted light at 4× magnification on a Nikon TE200 inverted microscope with a CCD camera (Quantifier, Optronics Inc.). Individual fields of view were montaged together (Photoshop CS2, Adobe Systems Inc.).

For analysis of lumen diameters from intact gracilis collateral whole mounts (i.e., vascular casting), collateral entrance regions were defined according to the following method. A cropped portion (560 µm × 560 µm) of the montaged image (previously randomized and de-identified) was taken of the collateral artery at the first visible branch point of a terminal arteriole from the primary collateral as it extended from either the muscular branch or saphenous artery as previously described (Heuslein et al., 2015b, 2016). After each cropped image region was taken, all images were randomized and de-identified. The mean diameter was then taken from 4 to 5 separate diameter measurements along the length of the cropped portion of the collateral artery. Mean collateral artery diameter was taken as the average of both the muscular and saphenous regions for a given mouse.

After imaging, muscles were rehydrated, cut, and paraffin embedded for cross-sectional analysis at the muscular branch and saphenous artery entrance regions to the collateral arteries. Resulting cross-sections were rehydrated and H&E stained for collateral artery structure analysis (day 21 post-FAL).

### Cross-sectional Analysis of Collateral Artery Structure

Sections (5-µm thickness) of paraffin embedded muscle from the muscular and saphenous regions were labeled for H&E. Individual fields of view encompassing the collateral vessels were imaged with a 40× water objective on a Zeiss inverted microscope (Zeiss Axioskop, Thornwood, NY, USA) with a CCD camera (Quantifier, Optronics Inc.). All images were randomized and de-identified before analysis. Lumen diameter, wall area, and wall thickness were determined using Fiji (Schindelin et al., 2012).

### Cross-sectional Analysis for Gastrocnemius Capillary Density

Sections (5-µm thickness) of paraffin embedded gastrocnemius muscles were deparaffinized, rehydrated, and blocked in Carbofree blocking solution (1:10, Vector Labs). Slides were then incubated with fluorophore conjugated primary antibody (isolectin-IB4-AlexFluor-647, 1:200, Life Technologies Inc.) overnight at 4°C. Nuclei were counterstained with Sytox green (500 µM, Life Technologies Inc.). Slides were washed and sealed with Prolong Gold (Life Technologies Inc.) to minimize photobleaching. Individual fields of view were imaged with a Nikon TE2000 C1 laser scanning confocal microscope with a 10× objective and the same imaging parameters for all fields of view. Fields of view were then montaged together using Fiji (Schindelin et al., 2012). Muscle areas were manually outlined and classified as either viable or non-viable tissue. The number of capillaries (Isolectin-B4^+^ vessels < 25 μm^2^ in diameter) and muscle area (identified from autofluorescence) were determined in each montaged image view using a semi-automated Fiji image analysis.

### Cross-sectional Analysis for Gastrocnemius Muscle Morphology

Sections (5-µm thickness) of paraffin embedded muscle from the gastrocnemius muscle were H&E labeled. Individual fields of view were imaged with a 10× objective on a Zeiss inverted microscope (Zeiss Axioskop) with a CCD camera (Quantifier, Optronics Inc.). Individual fields of view were montaged together (Photoshop CS2, Adobe Systems Inc.). All montaged images were randomized and de-identified before analysis. Muscle areas were manually outlined using Fiji (Schindelin et al., 2012). Tissue composition was classified into viable and non-viable, which were defined as:

1. Viable: fibers are present and have centrally located nuclei (regenerating) or fibers are comparable in size, organization, and structure to unligated control with peripheral nuclei (mature) (Meisner et al., 2014).
2. Non-viable: fibers lack nuclei, are rounded and dilated in appearance, have weak eosinophilic cytoplasm (necrotic) or where there is minimal presence of myoblasts and is dominated by fibrous matrix and adipose tissue (fibro-adipose) (Meisner et al., 2014).

### Statistical Analyses

All results are reported as mean ± SEM, unless otherwise noted. All data were first tested for normality and equal variance. Statistical significance was then assessed by a Student’s *t*-test or a two-way ANOVA followed by a Holm–Sidak multiple comparisons test. Data not following a normal distribution were tested for statistical significance by a Mann–Whitney *U*-test (GraphPad Prism 7.0). Significance was assessed at *p* < 0.05.

## Results

### miR-146a Inhibition Impairs Endothelial Tube Formation and Migration *In Vitro*

**Figure 1:**
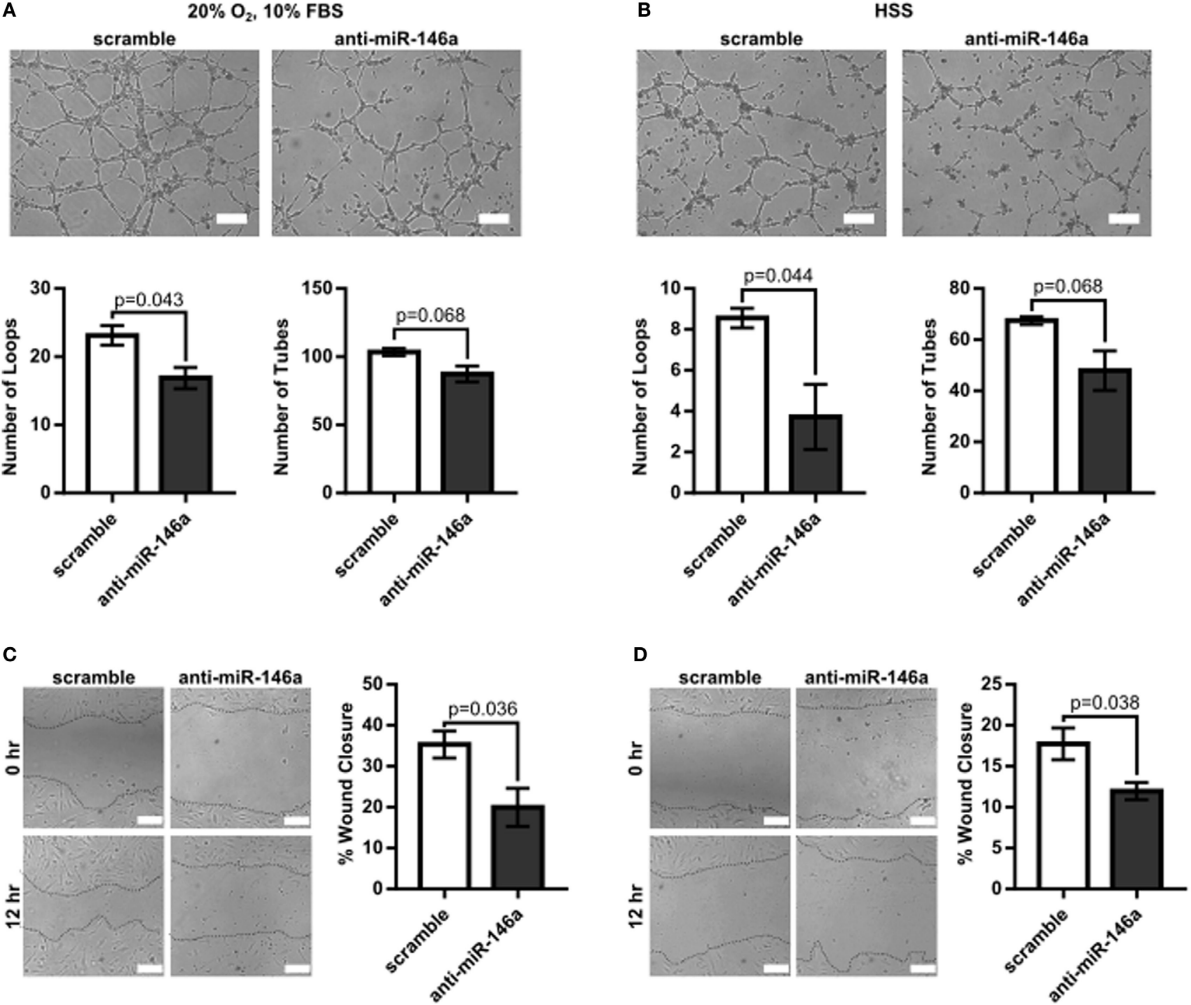
Impaired endothelial tube formation and migration with microRNA (miR)-146a knockdown *in vitro*. **(A,B)** Tube formation of human umbilical vein endothelial cells (HUVECs) transfected with 30 nM scramble or anti-miR-146a oligonucleotides after 6 h of exposure to growth [20% O_2_, 10% fetal bovine serum (FBS)] or hypoxia serum starvation (HSS) conditions (*n* = 3). **(C,D)** Endothelial wound closure in HUVECs after 12 h of exposure to either growth [**(C)**, *n* = 4] or HSS [**(D)**, *n* = 4] conditions. Student’s *t*-test. Data are mean ± SEM.

**Figure 2:**
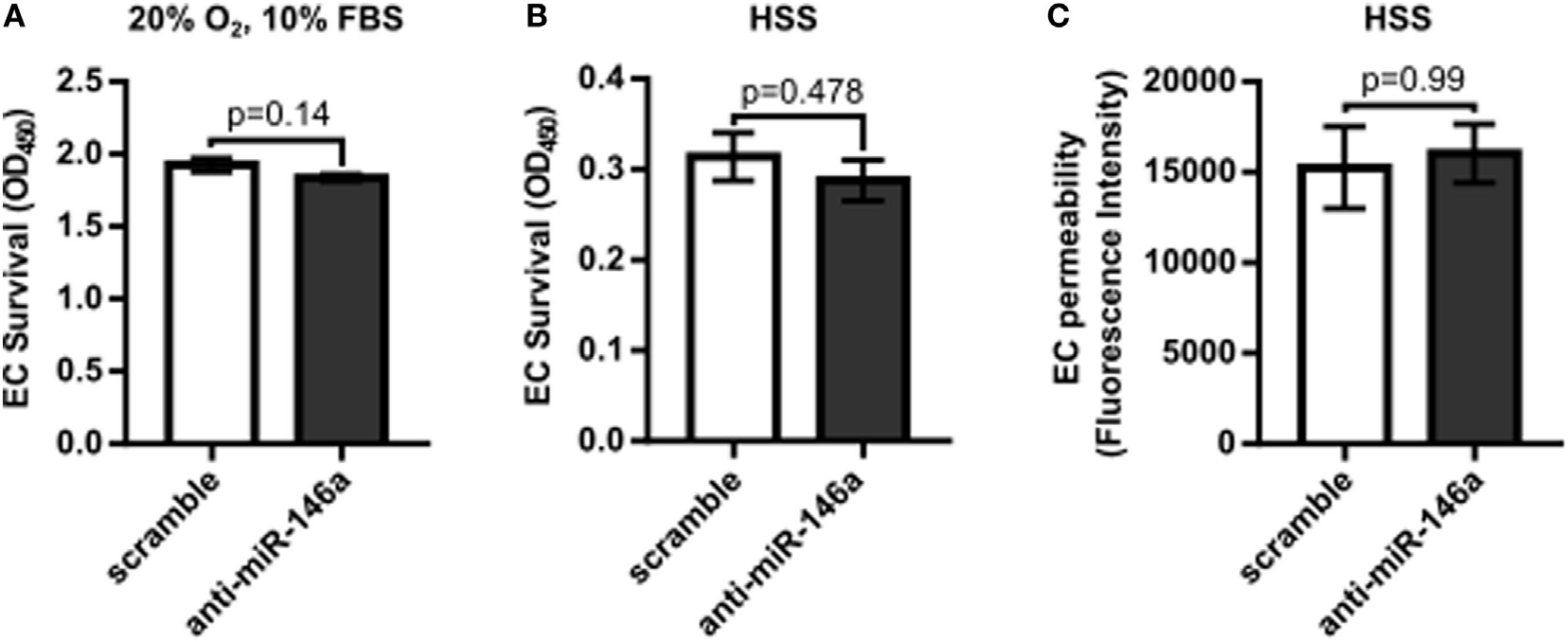
No significant effect on endothelial cell (EC) survival or permeability with microRNA (miR)-146a inhibition *in vitro*. **(A,B)** Twenty-four hours after transfection with 30 nM scramble or anti-miR-146a oligonucleotides, human umbilical vein endothelial cells (HUVECs) were exposed to 24 h of either growth [normoxia, 10% fetal bovine serum (FBS)] or hypoxia serum starvation (HSS) conditions. During the final 4 h, the tetrazolium salt WST-1 was added to the medium. Resulting salt cleavage to formazan by mitochondrial dehydrogenases was used as an indicator of cell viability (*n* = 4). Student’s *t*-test. **(C)** Transwell inserts (0.4 µm) were used to assess EC permeability *via* rhodamine dextran (70 kDa), after 24 h of HSS exposure in HUVECs transfected with scramble or anti-miR-146a oligonucleotides (*n* = 3). Mann–Whitney *U*-test. Data are mean ± SEM.

To first determine the effect of miR-146a inhibition on endothelial function, we investigated whether knockdown of miR-146a affected EC capillary-like tube formation, migration, survival, or permeability. HUVECs were transfected with 30 nM anti-miR-146a or scramble controls for 24 h before initiating the assay. Formation of capillary-like tube structures was assessed after 6 h of exposure to either normal growth (20% O_2_ and 10% FBS) or HSS (2% O_2_) conditions. ECs transfected with anti-miR-146a exhibited a significantly decreased number of EC loops and a trend toward decreased number of tubes compared to scramble in both growth and HSS conditions, suggesting an impact on EC network formation and motility (Figures 1A,B). This coincided with impaired EC migration with knockdown of miR-146a, as determined by an endothelial scratch assay, again in both normoxic growth (Figure 1C) and HSS (Figure 1D) conditions. There were no significant differences in EC survival or EC permeability between scramble and anti-miR-146a treated cells (Figure 2).

### Perfusion Recovery following FAL Is Improved by miR-146a Inhibition in Balb/c Mice

**Figure 3:**
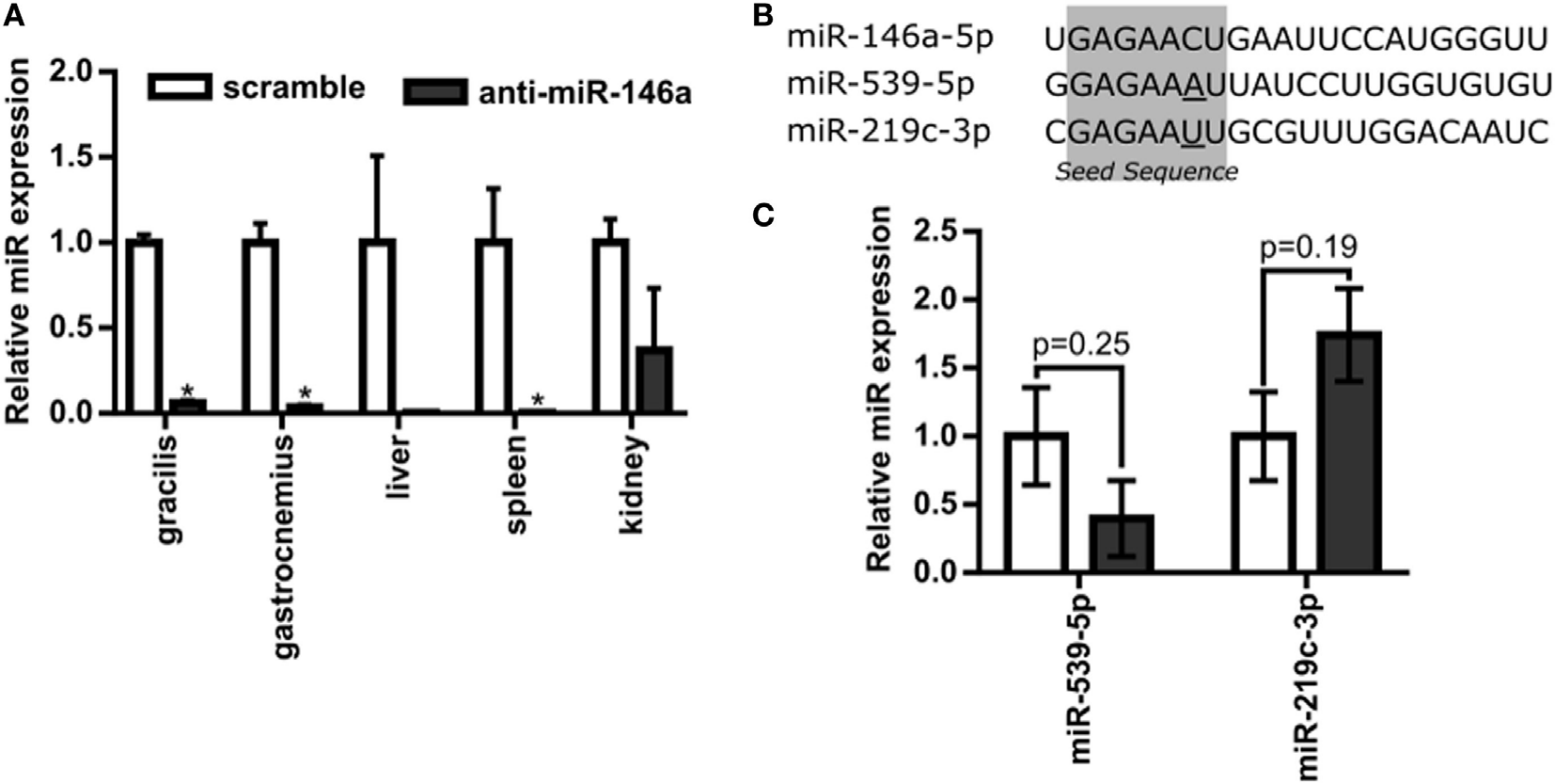
Systemic delivery of anti-microRNA (miR)-146a-5p with single intramuscular injection. **(A)** Relative miR-146a-5p expression in the gracilis and gastrocnemius muscles of ligated limbs as well as the liver, spleen, and kidney 7 days post-femoral arterial ligation (FAL) in Balb/c mice. Mice were treated with a single i.m. injection of 7.5 nmol scramble (*n* = 3) or anti-miR-146a (*n* = 4) locked nucleic acid oligonucleotides immediately post-FAL. *p < 0.05, Student’s *t-*test except kidney samples which were Mann–Whitney *U*-test. **(B)** Sequence of miR-146a-5p and two additional miRNAs with similar seed sequences, miR-539-5p and miR-219c-3p. Seed sequence is highlighted in gray with nucleotides altered from miR-146a-5p sequence underlined. **(C)** Expression of miR-539-5p and miR-219c-3p in gastrocnemius of ligated limb 7 days post-FAL (*n* = 3–4 for scramble and anti-miR-146a treated mice, respectively). Student’s *t-*test. Data are mean ± SEM.

**Figure 4:**
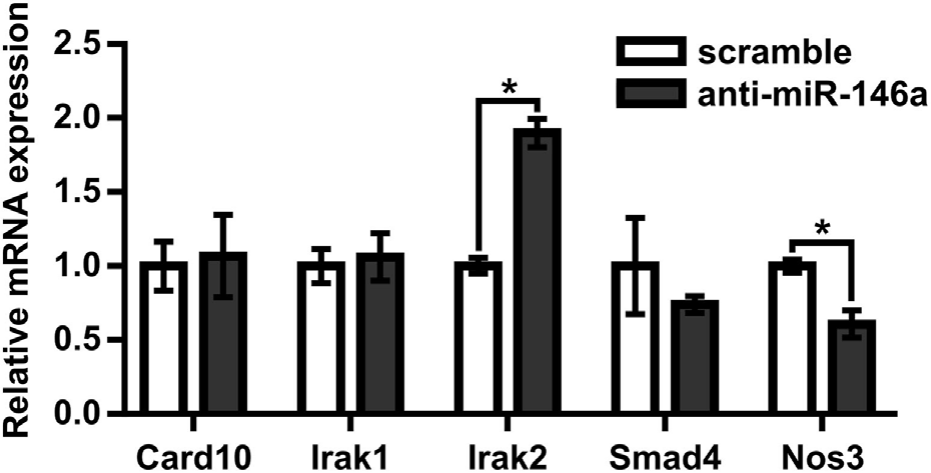
MicroRNA (miR)-146a regulates Irak2 and eNOS expression in ischemic gastrocnemius muscle. Relative mRNA expression of miR-146a targets in gastrocnemius muscle of ligated limb 7 days post-femoral arterial ligation in mice treated with scramble (*n* = 3) or anti-miR-146a (*n* = 4) oligonucleotide. *p < 0.05, Student’s *t*-test. Data are mean ± SEM.

Next, to test the hypothesis that miR-146a regulates perfusion recovery *in vivo*, we performed FALs on Balb/c mice immediately followed by intramuscular injection of scramble control or anti-miR-146a LNA oligonucleotides directly into each gracilis (i.e., thigh) muscle. We first assessed the efficacy of anti-miR-146a delivery. Local intramuscular injection substantially decreased miR-146a expression (>10-fold) in the gracilis muscle of the ligated limb 7 days post-FAL (Figure 3A). Interestingly, despite an intramuscular injection, anti-miR-146a treatment knocked down miR-146a expression in several other tissues including the gastrocnemius muscle (*p* < 0.001), spleen (*p* < 0.013), and liver (*p* = 0.06) (Figure 3A). Of note, miR-146a-5p differs in mature sequence from miR-146b-5p by only two nucleotides at their 3′ ends and they share identical seed sequences. We found that while miR-146a primers were specific (no cross-reactivity with miR-146b), miR-146b primers cross-reacted with miR-146a. As such, we could confidently determine miR-146a expression but could not confidently detect miR-146b due to this cross-reactivity. We therefore chose to determine the specificity of anti-miR-146a treatment by assessing the expression of two other miRs (miR-539-5p and miR-219c-3p) that differ in seed sequence from miR-146a-5p by only one nucleotide (Figure 3B). Expression of each of these miRs was not significantly altered by anti-miR-146a treatment (Figure 3C). We also assessed mRNA expression of several genes previously shown to be direct miR146a targets, including *Card10* (Rau et al., 2014), *Irak1* (Taganov et al., 2006), *Irak2* (Hou et al., 2009), and *Sirt4* to confirm functional knockdown of miR-146a. While it should be noted that these are at the transcript level, anti-miR-146a treatment increased expression of Irak2 as well as decreased eNOS (*Nos3*) mRNA expression, which has been previously shown to be indirectly regulated by miR-146a (Cheng et al., 2013), in the gastrocnemius muscle of anti-miR-146a treated mice (Figure 4).

**Figure 5:**
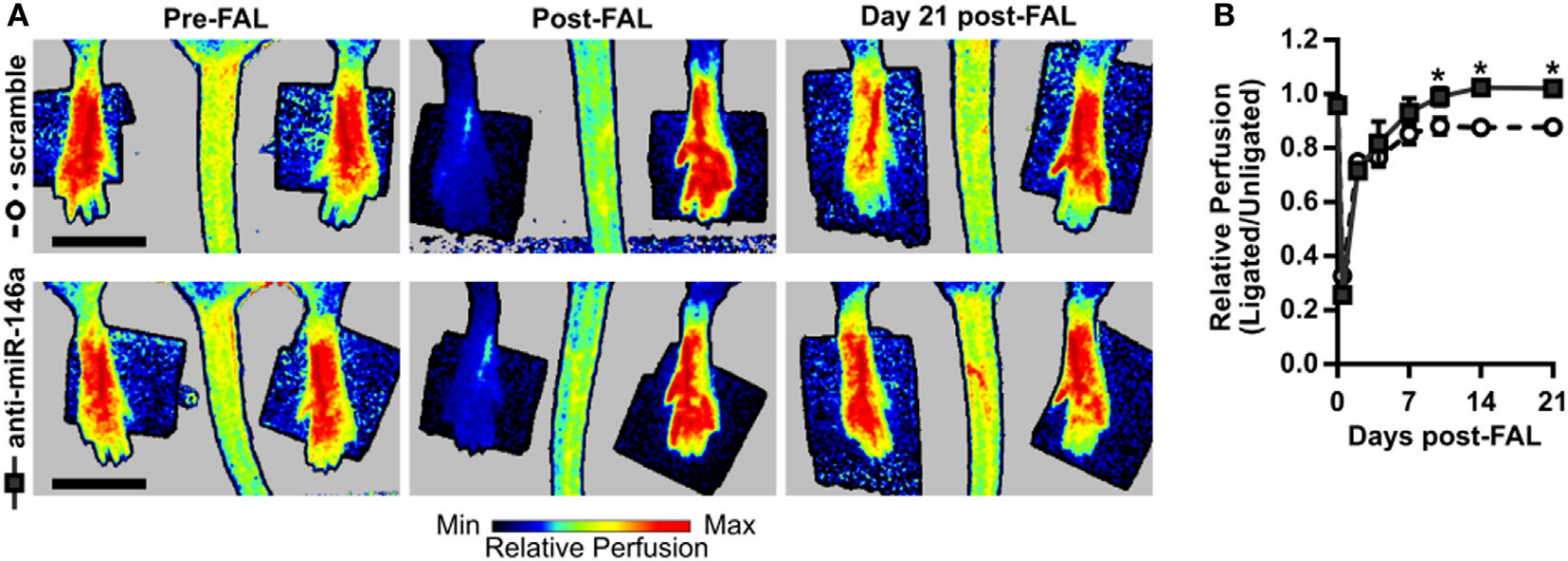
Improved perfusion recovery following femoral arterial ligation (FAL) in Balb/c mice with inhibition of microRNA (miR)-146a. **(A)** Representative images and **(B)** quantification of relative foot perfusion determined by laser Doppler perfusion imaging of mice treated with a single i.m. injection of 7.5 nmol scramble control (*n* = 6) or anti-miR-146a (*n* = 4) LNA oligonucleotides immediately post-FAL. *p < 0.05, two-way ANOVA with repeated measures followed by a Holm–Sidak multiple comparisons test. Data are mean ± SEM.

To next assess functional outcomes of anti-miR-146a treatment, we performed FALs and delivered anti-miR-146a or scramble control in a separate cohort of Balb/c mice followed by serial laser Doppler perfusion imaging. Laser Doppler measurements of the plantar surface of the foot indicated moderate ischemia immediately post-FAL, followed by an incomplete perfusion recovery in the scramble-treated control mice. However, anti-miR-146a treated mice exhibited an improved perfusion recovery compared to scramble as early as day 10 post-FAL. By day 21 post-FAL, anti-miR-146a treated mice demonstrated a 16% increase in perfusion recovery vs. scramble-treated controls (Figures 5A,B).

### miR-146a Inhibition Does Not Regulate Capillary Density following FAL in Balb/c Mice

**Figure 6:**
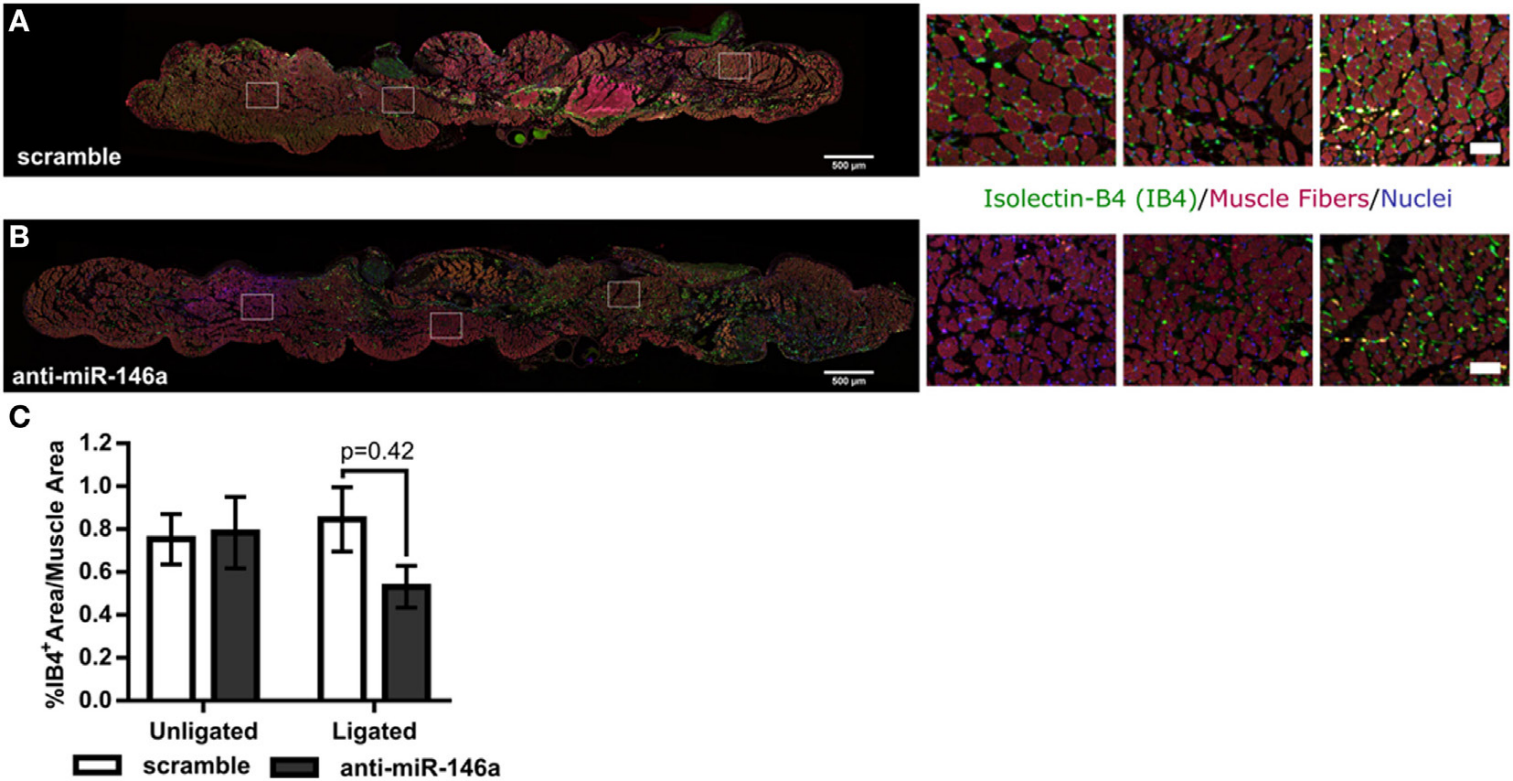
MicroRNA (miR)-146a inhibition does not improve capillary density following femoral arterial ligation (FAL) in Balb/c mice. **(A,B)** Representative images of gastrocnemius muscle labeled for capillaries (isolectin-b4, green), muscle fibers (autofluorescence, red), and nuclei (SYTOX, blue) from ligated limbs 21 days post-FAL. Scale bar = 500 μm. Insets highlight heterogeneous capillary density in viable muscle of anti-miR-146a treated mice. Scale bar = 50 μm. **(C)** Bar graph of percent isolectin-b4 (IB4) positive area per muscle fiber area over entire muscle in ligated and unligated gastrocnemius muscles 21 days post-FAL. *n* = 5 for scramble and *n* = 4 for anti-miR-146a groups.

To determine the mechanism of increased perfusion recovery with anti-miR-146a treatment, we first examined if miR-146a inhibition altered capillary density in the gastrocnemius muscle following FAL. Gastrocnemius muscle cross-sections were labeled using SYTOX green as a nuclear marker and isolectin-B4 as an EC marker. Muscle area autofluorescence was used to determine muscle area (Figures 6A,B). We found that there was no significant difference in capillary density determined by percentage of isolectin-B4-positive area per muscle area between anti-miR-146a and scramble-treated mice (Figure 6C).

### miR-146a Inhibition Enhances Arteriogenesis following FAL

**Figure 7:**
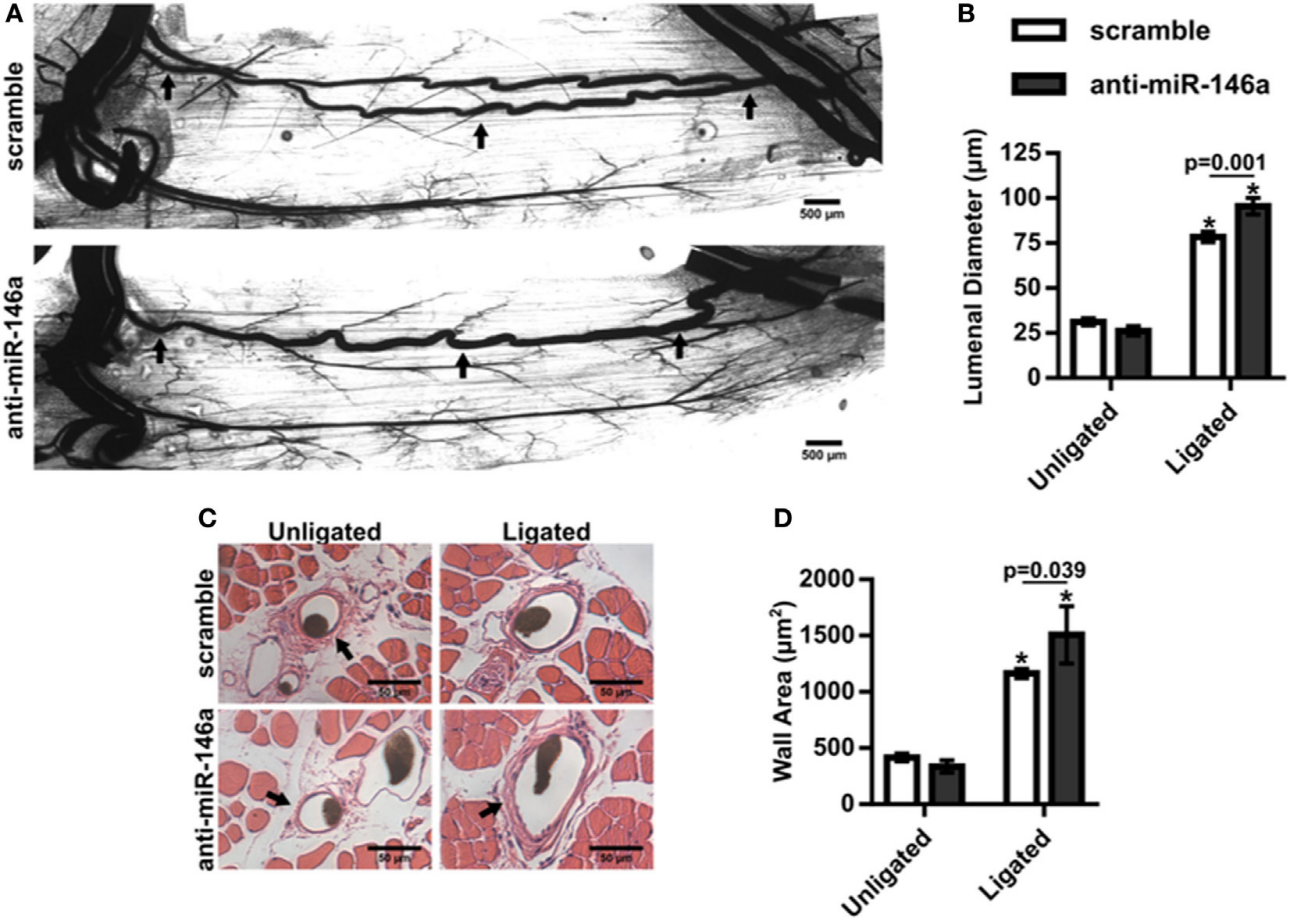
Inhibition of microRNA (miR)-146a enhances collateral artery growth following femoral arterial ligation (FAL). **(A)** Representative whole mount vascular cast images of gracilis collateral arteries 21 days post-FAL in scramble (top) or anti-miR-146a (bottom) treated Balb/c mice. Scale bar = 500 µm. **(B)** Bar graph of mean lumenal diameter along collateral artery length for ligated and unligated limbs of anti-miR-146a (*n* = 4) and scramble-treated mice (*n* = 6). *p < 0.001 vs. unligated, two-way ANOVA followed by Holm–Sidak test for multiple comparisons. **(C)** Representative H&E stained cross-sections of collateral arteries from ligated and unligated limbs. Scale bar = 50 µm. **(D)** Bar graph of wall area from H&E stained cross-sections (*n* = 4 or 6 for anti-miR-146a and scramble-treated mice, respectively). *p < 0.05 vs. unligated, two-way ANOVA followed by Holm–Sidak test for multiple comparisons. Arrows in **(A,C)** indicate primary collateral artery. Data are mean ± SEM.

**Figure 8:**
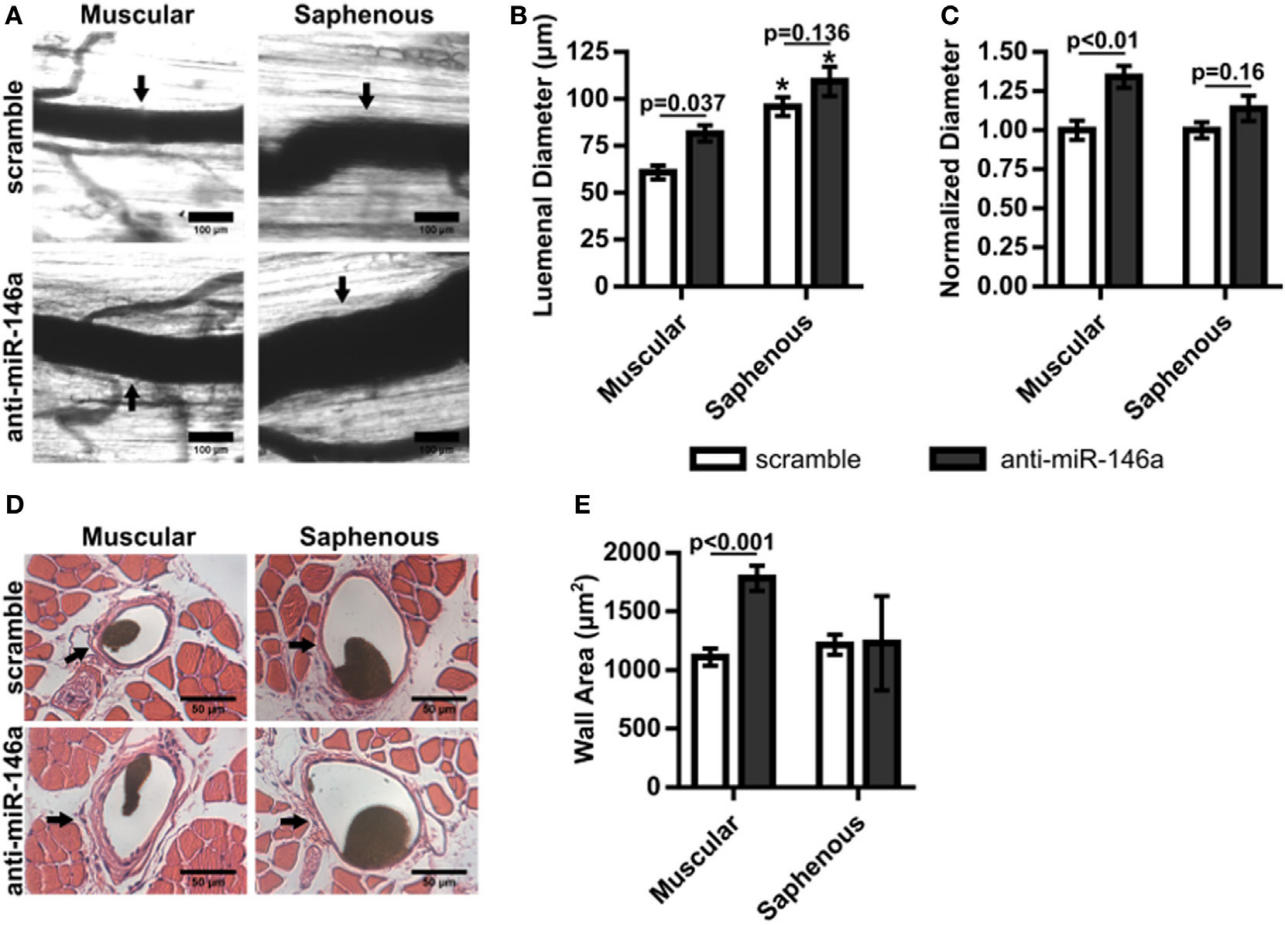
Regional analysis of gracilis collateral arteries. **(A)** Representative vascular cast images from muscular and saphenous collateral artery segments 21 days post-femoral arterial ligation (FAL) in scramble and anti-microRNA (miR)-146a treated Balb/c mice. **(B)** Bar graph of lumenal diameter post-FAL from ligated limb of mice treated with non-targeting control in each region (*n* = 4 or 6 for anti-miR-146a and control-treated mice, respectively). *p < 0.05 vs. muscular region for a given treatment, two-way ANOVA followed by Holm–Sidak test for multiple comparisons. **(C)** Lumenal diameters normalized to the ligated limb of mice treated with scramble in each region (*n* = 4 or 6 for anti-miR-146a and control-treated mice, respectively). Student’s *t*-test. **(D)** Representative H&E stained cross-sections from muscular and saphenous collateral artery segments 21 days post-FAL. **(E)** Bar graph of wall area from H&E stained cross-sections (*n* = 4 or 6 for anti-miR-146a and scramble-treated mice, respectively). Two-way ANOVA followed by Holm–Sidak test for multiple comparisons. Arrows indicate the primary collateral artery in **(A,D)**. Data are mean ± SEM.

**Figure 9:**
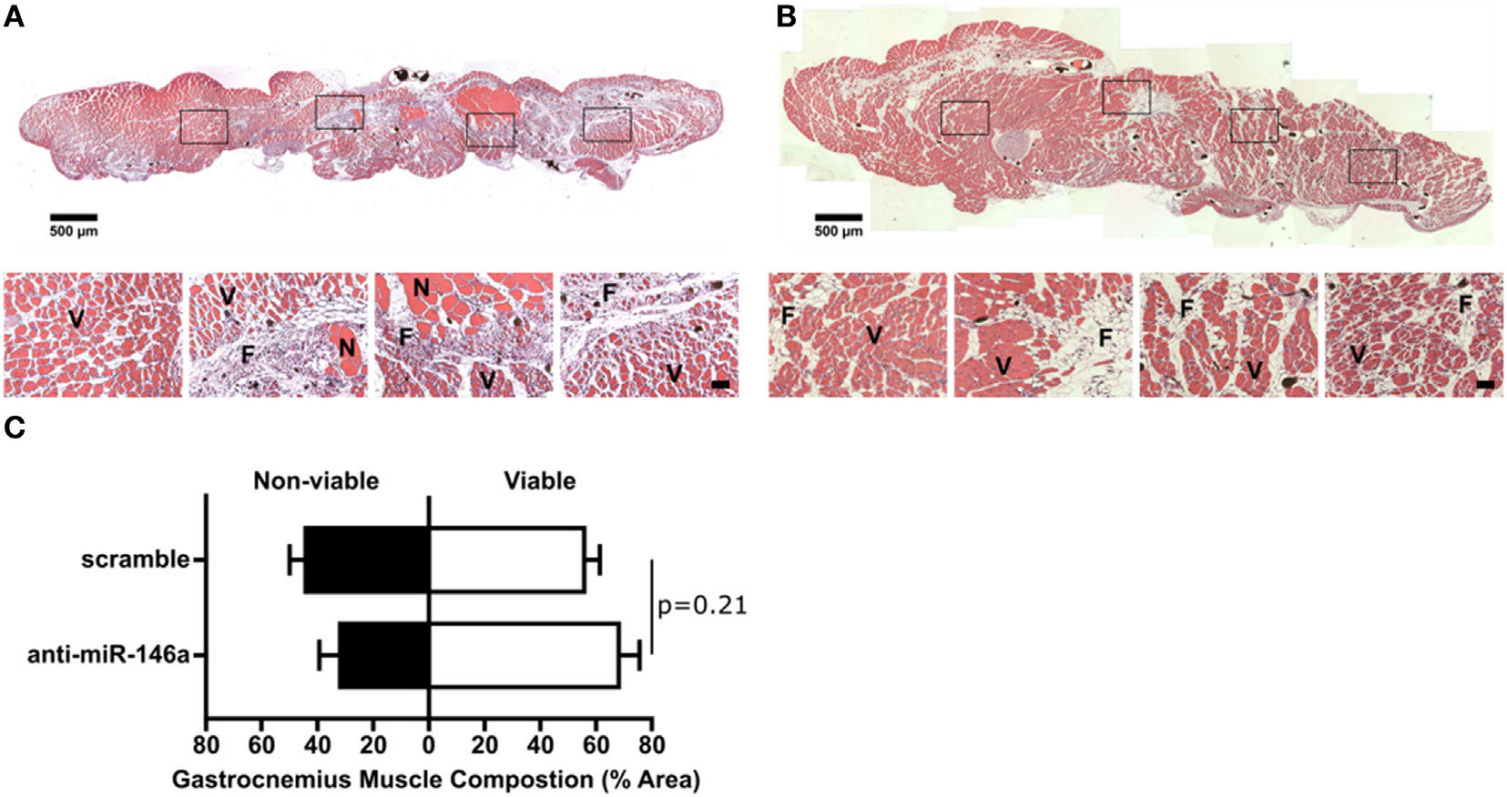
Effect of single dose of anti-microRNA (miR)-146a to gracilis muscle on gastrocnemius muscle composition. **(A,B)** Representative images of H&E staining of gastrocnemius muscle for ligated limb of Balb/c mice treated with scramble **(A)** or anti-miR-146a **(B)** locked nucleic acid oligonucleotide immediately after femoral arterial ligation (FAL) (scale bar = 500 μm, inset scale bar = 50 μm). V, viable muscle; N, necrotic tissue; F, fibro-adipose tissue. **(C)** Bar graph of the percentage of gastrocnemius muscle that is viable (white) or non-viable (black) at day 21 post-FAL in each group (*n* = 4–5 for anti-miR-146a and scramble groups, respectively). Student’s *t*-test. Data are mean ± SEM.

We next examined whether miR-146a inhibition affected arteriogenesis by measuring luminal diameter growth of gracilis collateral arteries 21 days post-FAL (Figure 7A). Both control and anti-miR-146a treated mice experienced significant (*p* < 0.001) arteriogenesis in the ligated limb compared to sham-operated (unligated) controls. However, arteriogenesis was further enhanced with miR-146a inhibition, as collateral artery diameter was 22% greater than in controls (Figures 7A,B). Furthermore, cross-sectional analysis of these collateral arteries was used to determine vessel wall area, demonstrating increased wall area in anti-miR-146a treated mice compared to control (Figures 7C,D). As we have previously shown that the extent of arteriogenesis varies depending on the flow waveform following FAL (Heuslein et al., 2015b), we analyzed both the muscular (non-reversed flow) and saphenous (reversed flow) collateral segments to determine if miR-146a inhibition led to differences in regional growth. Anti-miR-146a treated mice exhibited a ~35% increase in lumen diameter in muscular (non-reversed flow) segments compared to controls while there was no significant difference in saphenous (reversed flow) segments (Figures 8A–C). Wall area was also significantly greater in muscular (non-reversed flow) segments in anti-miR-146a treated mice while there was no significant difference in saphenous (reversed flow) regions compared to controls (Figures 8D,E). Finally, we examined the effect of altered miR-146a expression on the composition of ischemic muscle tissue downstream of the femoral artery occlusion. Gastrocnemius muscle tissue was categorized as viable (mature and regenerating fibers) or non-viable (necrotic and fibro-adipose tissue) by histological analysis 21 days after FAL (Figure 9). There was no significant difference (*p* = 0.21) in muscle composition (i.e., increased viable tissue) between scramble and anti-miR-146a treated mice.

## Discussion

In this study, we sought to determine if miR-146a regulates angiogenesis, arteriogenesis, and perfusion recovery following FAL. We first assessed its role in endothelial function and angiogenesis *in vitro* by inhibiting miR-146a in HUVECs and assessing capillary-like tube formation, migration, survival, and permeability. Knockdown of miR-146a attenuated both network-like formation and EC migration while there was no effect on EC survival or permeability. These data would suggest that miR-146a would have no beneficial effect on angiogenesis. To next determine the role of miR-146a *in vivo*, we delivered a single, intramuscular injection of anti-miR-146a LNA oligonucleotides immediately following FAL in Balb/c mice and assessed perfusion recovery, angiogenesis, and arteriogenesis. While control-treated mice exhibited impaired perfusion recovery, miR-146a inhibition elicited a significantly improved recovery. Interestingly, miR-146a inhibition did not significantly affect capillary density or gastrocnemius muscle composition. However, anti-miR-146a was able to significantly enhance collateral artery growth. Together, our results indicate that miR-146a regulates perfusion recovery *via* arteriogenesis without a significant impact on angiogenesis.

Given our previous results in which miR-146a expression was decreased in ECs exposed to an “amplified arteriogenesis” flow waveform, we would have hypothesized that miR-146a inhibition would lead to enhanced collateral artery growth and thus enhanced perfusion recovery (Heuslein and Price, 2017). However, the role of miR-146a in angiogenesis was less clear. One previous report found that inhibition of miR-146a led to increased capillary sprouting in both aortic ring and laser-induced choroidal neovascularization assays (Halkein et al., 2013). In contrast, several previous studies have found that loss of miR-146a impairs angiogenesis *in vitro* (Zhu et al., 2013, 2016; Rau et al., 2014; Li et al., 2017), which would suggest miR-146a inhibition should impair perfusion recovery. In our hands, *in vitro* miR-146a inhibition attenuated both network-like formation and EC migration while there was no effect on EC survival or permeability. Of note, our assessments of EC function *in vitro* were performed at single time points based on similar studies (Hazarika et al., 2013; Zhu et al., 2013; Rau et al., 2014; Wang et al., 2015). Thus, they do not capture the dynamics of anti-miR-146a treatment. Despite this potential limitation, our *in vitro* results are consistent with later studies wherein miR-146a inhibition attenuated angiogenesis and EC migration *in vitro* (Zhu et al., 2013, 2016; Rau et al., 2014; Li et al., 2017).

We then moved to test the effect of miR-146a inhibition *in vivo* following FAL. Despite the impaired angiogenesis we observed *in vitro*, miR-146a inhibition led to a significantly enhanced perfusion recovery in Balb/c mice *in vivo*. Perfusion recovery following FAL can be dependent on angiogenesis (Meisner et al., 2013), tissue clearance and skeletal muscle regeneration (Meisner et al., 2014), and/or arteriogenesis. To this end, we found no significant difference in capillary density in the gastrocnemius muscle of FAL-operated limbs between anti-miR-146a and scramble-treated mice with a possible trend toward decreased capillary density in anti-miR-146a treated mice. This corresponded to decreased gastrocnemius eNOS expression in FAL-operated limbs (Figure 4), which was previously shown to be an indirect target of miR-146a (Cheng et al., 2013), though this was only assessed at the mRNA level whereas phosphorylated eNOS is the main effector of eNOS function. Moreover, there was no significant difference in gastrocnemius muscle composition between anti-miR-146a and scramble control-treated mice. However, mice treated with anti-miR-146a exhibited significantly enhanced collateral artery growth, corresponding to the observed increase in foot perfusion in these mice. Our *in vivo* results indicate that miR-146a regulates perfusion recovery *via* arteriogenesis without a significant impact on angiogenesis.

The lack of observed angiogenesis and muscle regeneration may have several alternative explanations. In this study, capillary density was only examined at day 21. While earlier time points would capture dynamic differences in capillary density between the groups, we chose day 21 as a better representation of the more clinically relevant, steady-state condition. In addition, while anti-miR-146a delivery appeared to systemically inhibit miR-146a expression, angiogenesis is driven by ischemia in the downstream gastrocnemius (i.e., calf) muscle and is a later response to FAL than arteriogenesis. It is therefore possible that a different anti-miR-146a dosing regimen (e.g., timing, amount, number of injections, etc.) may be required to directly induce angiogenesis in addition to arteriogenesis. Furthermore, angiogenesis can also be indirectly induced if the increased blood flow due to arteriogenesis is sufficient to stimulate capillary sprouting. The relatively modest increase in perfusion with anti-miR-146a may not be sufficient to induce additional angiogenesis in this mildly ischemic ligation scheme as opposed to other ligation/excision surgical models which induce significantly greater ischemia in the gastrocnemius. Finally, miR-146a mediated inflammation may lead to different outcomes for arteriogenic and angiogenic processes (i.e., anti-miR-146a be pro-arteriogenic while having no or anti-angiogenic effects in the ischemic gastrocnemius).

MicroRNA-146a’s known role as a critical negative regulator of inflammation may help explain its effect on arteriogenesis. In the presence of an inflammatory stimulus (i.e., IL-1β), miR-146a inhibition increases NFκB activity, adhesion molecule (i.e., ICAM-1, VCAM-1) expression, and monocyte adhesion to ECs, whereas miR-146a overexpression blunts EC activation (Cheng et al., 2013). We previously demonstrated a similar phenotype in ECs exposed to pro-arteriogenesis flow waveforms. ECs exposed to a reversed flow (“amplified arteriogenesis”) waveform exhibit decreased miR-146a expression as well as increased NFκB activity, ICAM-1 expression, and monocyte adhesion, whereas EC activation is attenuated when exposed to a non-reversed (“moderate arteriogenesis”) flow waveform (Heuslein et al., 2015b). Importantly, NFκB signaling is well known to be necessary for arteriogenesis (Tirziu et al., 2012; Sweet et al., 2013) through ICAM-1 (Lan et al., 1994; Nagel et al., 1994) dependent monocyte/macrophage recruitment (Hoefer et al., 2004). Our results are, therefore, consistent with the hypothesis that miR-146a inhibition leads to enhanced arteriogenesis, predominantly in non-reversed flow (i.e., muscular) collateral segments, *via* upregulation of pro-inflammatory (i.e., NFκB) EC activation.

In addition to ECs, miR-146a is also a well-known regulator of inflammatory signaling in leukocytes (Taganov et al., 2006; Boldin et al., 2011; Zhao et al., 2011; Etzrodt et al., 2012). A previous study demonstrated that miR-146a selectively controlled the amplitude of the Ly6C^hi^ monocyte response during an inflammatory challenge. To this end, loss of miR-146a increased Ly6C^hi^ monocyte proliferation and migration, whereas it did not affect Ly6C^lo^ monocytes (Etzrodt et al., 2012). Following FAL, inflammatory monocytes (Ly6C^hi^/CX3CR1^lo^/CCR2^hi^) are recruited to sites of active inflammation, leading to a perivascular accumulation of monocytes/macrophages that is critical for arteriogenesis (Ito et al., 1997; Heil et al., 2004; Shireman, 2007; Capoccia et al., 2008; Nickerson et al., 2009b; Meisner and Price, 2010; Troidl et al., 2013). Our anti-miR-146a intramuscular injections do result in a systemic knockdown of miR-146a and are not cell-specific. It is, therefore, possible that the observed anti-miR-146a mediated enhancement in arteriogenesis is also mediated *via* pericollateral Ly6C^hi^ monocyte proliferation and/or migration.

MicroRNA-146a inhibition is able to stimulate arteriogenesis and improve perfusion recovery; however, given its inability to stimulate angiogenesis and muscle regeneration, its utility as a clinical therapeutic is unclear. Indeed, while it is important to restore the driving pressure to the distal tissue *via* lumen expansion of collateral arteries bypassing the occlusion(s) (i.e., arteriogenesis) in PAD patients (Meisner et al., 2013, 2014; Heuslein et al., 2015a), it is also imperative to stimulate angiogenesis, as capillary density is reduced in these patients (Duscha et al., 2011; Robbins et al., 2011; Annex, 2013). To this end, improved functional outcomes induced by supervised exercise in PAD patients with intermittent claudication are preceded by and correlated with improvement in capillary density within ischemic muscle (Duscha et al., 2011; Robbins et al., 2011). Therapies that utilize a combination of microvascular expansion (i.e., angiogenesis) and restoration of large vessel flow (i.e., stenting, arteriogenesis) are more likely to be an “optimal” treatment (Heuslein et al., 2015a). Further supporting this hypothesis, the ERASE trial found that a combination therapy of revascularization and exercise improved functional outcomes of PAD patients compared to just exercise alone (Fakhry et al., 2015). Overexpression of miR-93 (Ganta et al., 2017) or miR-199a inhibition (Heuslein et al., 2017) have both been shown to be capable of inducing arteriogenesis and improving in the ischemic downstream tissue, demonstrating such miR-based approaches are indeed possible.

In addition, miR-146 inhibition may not be an ideal therapeutic as it could elicit the so-called “Janus phenomenon,” wherein pro-arteriogenic therapies also promote atherosclerosis (Epstein et al., 2004). To this end, one study found elevated miR-146a to be atheroprotective, as systemic delivery of miR-146a mimics in Apoe^−/−^Ldlr^−/−^ and Ldlr^−/−^ mice attenuated monocyte/macrophage activation and atherosclerosis (Li et al., 2015). Furthermore, local delivery of anti-miR-146a increased neointimal formation following carotid artery injury in the mouse (Chen et al., 2015). However, miR-146a inhibition may have differential effects on plaque formation depending on the specific cell type. Deletion of endogenous miR-146a within BM-derived cells reduced atherosclerotic plaque formation, whereas miR-146a deletion in the vasculature enhanced EC activation and atherogenesis (Cheng et al., 2017). Future work could determine the cell-specific effect of miR-146a inhibition on arteriogenesis, perfusion recovery, and atherosclerotic plaque stability in an experimental model of PAD with advance atherosclerosis.

## Ethics Statement

This study was carried out in accordance with the recommendations of the American Heart Association Guidelines for the Use of Animals in Research. The protocol was approved by the Institutional Animal Care and Use Committee at the University of Virginia (Protocol 3814).

## Author Contributions

JH designed and conducted the experiments, analyzed data, and wrote the manuscript. SM and JS helped conduct the experiments and analyze the data. BA and RP supervised the overall study and analyzed data. All authors read and edited the manuscript.

## Conflict of Interest Statement

The authors declare that the research was conducted in the absence of any commercial or financial relationships that could be construed as a potential conflict of interest.
